# Novel *GRIA2* variant in a patient with atypical autism spectrum disorder and psychiatric symptoms: a case report

**DOI:** 10.1186/s12887-022-03702-7

**Published:** 2022-11-03

**Authors:** Qianyun Cai, Zhongjie Zhou, Rong Luo, Tao Yu, Dengfeng Li, Fan Yang, Zuozhen Yang

**Affiliations:** 1 Department of Pediatrics, West China Second University Hospital, Sichuan University, Chengdu, 610041 Sichuan China; 2 Key Laboratory of Obstetric & Gynecologic and Pediatric Diseases and Birth Defects of Ministry of Education, Sichuan University, Chengdu, 610041 Sichuan China; 3 Department of Orthopedics, West China Hospital, Sichuan University, Chengdu, 610041 Sichuan China; 4 Cipher Gene LLC, Beijing, 100089 China

**Keywords:** Whole-exome sequencing, GRIA2, AMPA receptor, ASD, Case report

## Abstract

**Background:**

As sequencing technology has advanced in recent years, a series of synapse-related gene variants have been reported to be associated with autism spectrum disorders (ASDs). The α-amino-3-hydroxy-5-methyl-4-isoxazole propionic acid (AMPA) receptor is a subtype of the ionotropic glutamate receptor, whose number or composition changes can regulate the strength and plasticity of synapses.

**Case presentation:**

Here, we report a de novo* GRIA2* variant (NM_001083619.3: c.2308G > A, p.Ala770Thr) in a patient with obvious behavior regression and psychiatric symptoms. It encodes GluA2, which is the crucial subunit of the AMPA receptor, and the missense variation is predicted to result in instability of the protein structure.

**Conclusions:**

The association between *GRIA2* variants and onset of ASD symptoms is rare, and our study expands the spectrum of phenotypic variations. For patients with an unexplained etiology of ASD accompanied by psychiatric symptoms, genetic causes should be considered, and a complete genetic evaluation should be performed.

**Supplementary Information:**

The online version contains supplementary material available at 10.1186/s12887-022-03702-7.

## Background

Autism spectrum disorders (ASDs) are a group of neurological development disorders that are characterized by deficits in social interaction and stereotyped behaviors [1]. The prevention of ASDs has become a top priority in the current public health field [2]. Parents usually notice abnormal language and behavior when their children were two or three years old [3]. However, some children with ASD have a normal or near-normal early stage of development and then develop one or more idiosyncratic features of ASD, such as language regression [4]. Furthermore, the development and performance of symptoms vary widely among individuals [5]. To meet the individual diagnosis, treatment, and prognosis needs of patients with ASD, complete genetic evaluation has become one of the critical methods.

With the advancement of sequencing technology, genome and exome sequencing in patients with ASDs has reached an unprecedented scale in the last two years [6, 7]. A recent study showed that AMPA receptor GluA2 subunit defects are a cause of neurodevelopmental disorders, including ASDs [8]. The AMPA receptor is one of the ionotropic glutamate receptors and is the major mediator of fast excitatory neurotransmission in the vertebrate brain [9]. GluA2 subunit encoded by the *GRIA2* gene has a crucial role in the regulation of AMPA receptor Ca^2+^ permeation and voltage rectification. This is largely mediated by the arginine residues in the ion-selectivity filter, which is produced by the posttranscriptional editing of the glutamine codon (CAG- > CGG; Q- > R) [10]. Additionally, mouse Q/R site point mutation of the *Gria2* gene had an ~ 20% reduction in GRIA2 RNA editing, and exhibited loss of dendritic spines, hippocampal CA1-neuron loss, and learning and memory impairments [11]. These studies reflect the important role of the *GRIA2* gene in neurodevelopment; however, there are few reports of *GRIA2* variations in neurological diseases were shown.

Here, we report a four-year-old girl who was affected by decreased verbal expression, visual interaction, and interaction with her family. ASD-like features and neuropsychiatric symptoms were observed; we also found a de novo variant in the *GRIA2* gene (NM_001083619.3: c.2308G > A, p.Ala770Thr) by whole-exome sequencing (WES). This is the third study on the *GRIA2* variant associated with neurodevelopmental disorders, and our study expands the spectrum of phenotypic variations of GRIA2.

## Case presentation

### Case report

Our patient was a four-year-old girl who came to the hospital with autism-like manifestations and psychiatric symptoms for 2 months. She exhibited severely decreased verbal expression, eye contact and social interaction. She spent most of her time watching cartoons, and she had visual hallucinations in which she could see the leopard from the cartoon. She also talked to herself and had the stereotyped behavior of rubbing her hands and feet. She cried and screamed when her requests were not met. She rarely communicated with family members or friends, and her response to painful stimuli was significantly reduced. During the course, there were no fevers, seizures, disturbances of consciousness, or movement disorders. The patient was delivered by cesarean section at 38 weeks of gestation with a birth weight of 3500 g. The perinatal period was uneventful. There was no history of hypoxic asphyxia or postnatal resuscitation. Her growth, language, motor abilities, and social interaction were considered normal before. She could speak at one year old and could walk without support at the age of one year and four months old. She could also sing and recite English rhymes before. Her mother denied hereditary diseases in the family and other special medical histories. According to the DSM-5 criteria, she met the diagnosis of ASD, and the severity assessments in both domains of social communication/interaction and repetitive/restricted behavior were grade 3.

**Fig. 1:**
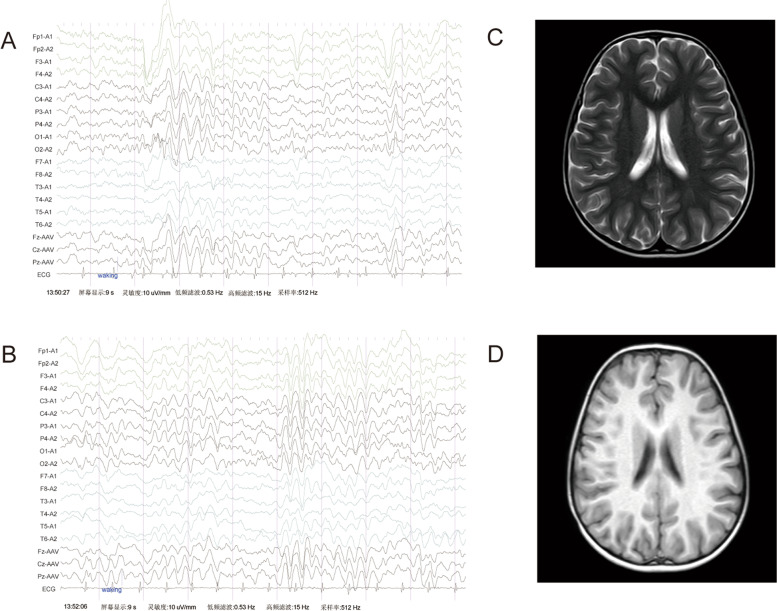
VEEG and MRI result in our patient. VEEG results showed medium-amplitude 3.5–4.5 Hz slow waves in the central, parietal, occipital, and posterior temporal regions **A** or all leads **B**. In addition, the background rhythm was normal and no epileptic discharges were detected. Axial T2 weighted **C** and T1 weighted **D** images of brain MRI from the patient were normal

Denver Developmental Screening Test (DDST) results indicated that she was abnormal in all four (fine motor, gross motor, personal-social, and language) areas, and her language development was equivalent to that of a child aged 19 months. The autism behavior checklist (ABC) showed a positive result. Video electroencephalogram (VEEG) results showed that the background rhythm of the child was normal and no epileptic discharges were detected. The patient’s VEEG results showed medium-amplitude slow waves in the central, parietal, occipital, and posterior temporal regions (Fig. 1A) or all leads (Fig. 1B) in the awake state. VEEG results in the sleep state were normal. Brain magnetic resonance imaging (MRI) (Fig. 1C, D), routine blood examination, liver function, kidney function, electrolyte levels, blood ammonia levels, lactic acid levels, and pyruvic acid levels were normal. Serum anti-N-methyl-d-aspartate (NMDA) receptor antibody IgG + 1:10, and other autoimmune encephalitis-related antibody levels were normal. The blood paraneoplastic syndrome-related antibody, aquaporin-4, and myelin oligodendrocyte glycoprotein levels were normal. Cerebrospinal fluid autoimmune encephalitis related antibodies, paraneoplastic syndrome-related antibodies, and oligoclonal bands were normal.

Considering the possibility of autoimmune encephalitis, she was treated with gamma globulin and methylprednisolone pulse therapy successively, but her condition did not improve significantly. After discharge, with the improvement of family relationships, enhanced parent–child companionship, and treatment with risperidone, she had a more stable mood than before. She played, watched fewer cartoons, and had some verbal communication with her family. However, she still had repetitive stereotyped behaviors, such as hand-wringing.

### Identification of the de novo variant in *GRIA2*

**Fig. 2:**
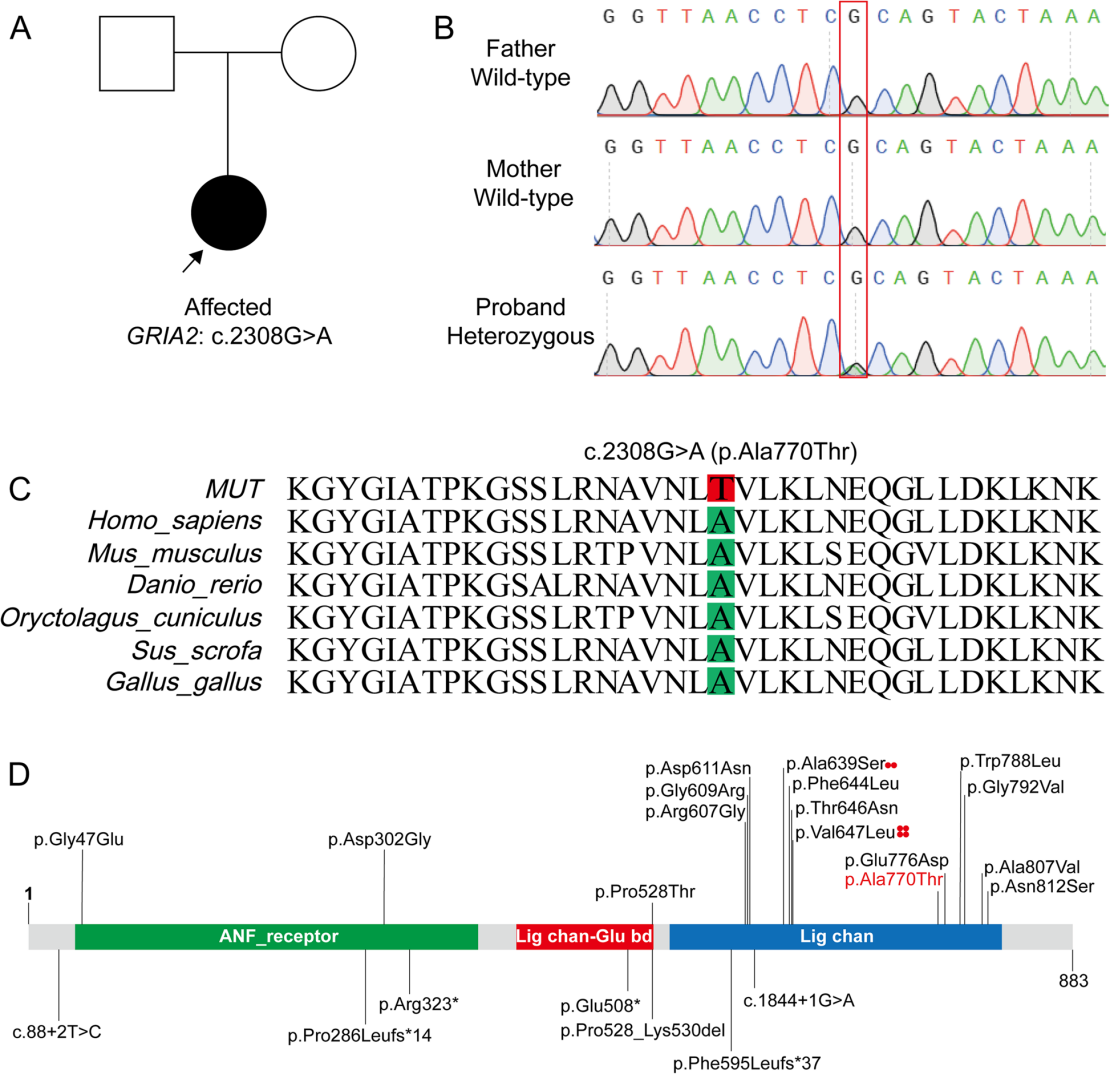
Identification of de novo variant in *GRIA2* gene. **A** The pedigree of this family. The affected subject is indicated by a filled symbol with an arrow. **B** Sanger sequencing of *GRIA2* in the trio family. The c.2308G > A was found and confirmed in proband indicated by the red box. **C** Conservation of A770 across multiple species. The targeted residues were highlighted with red (wild-type) and green (mutated) boxes. **D** Schematic representation of *GRIA2* variants. Missense variants are marked at the top of the diagram, splice and truncated variants are below. Three domains are shown in protein: ANF receptor (41-381aa, receptor family ligand binding region), Lig chan-Glu bd (414-529aa, ligated ion channel L glutamate, and glycine-binding site), Lig chain (543-824aa, ligand-gated ion channel)

To further investigate the cause of the disease, a trio-WES was performed using peripheral blood samples from the patient and her parents. A de novo variant (NM_001083619.3: c.2308G > A) in *GRIA2* was predicted to change the 770^th^ amino acid from alanine to threonine (p.Ala770Thr). This heterozygous variant was not inherited from her parents and was confirmed by Sanger sequencing (Fig. 2A, B).

This novel c.2308G > A in *GRIA2* was predicted to cause disease by bioinformatic tools (Table 1), and it was also absent from the genome aggregation database (gnomAD), the Exome aggregation consortium (ExAC), and the 1000 genome databases, which explains the rarity of this variant. According to the ACMG guidelines, it was rated as a variant of uncertain significance (*VUS*) through PS2_Moderate + PM2_Supporting + PP3. There are 84 variants in *GRIA2* contained in the ClinVar dataset, and only 46 are single nucleotide variations (SNVs). The variant in our patient was also not found in ClinVar.

**Table 1:** Variant information

Gene	Variant	Inheritance	MAF			Variants hazard prediction		
			ExAc	gnomAD	1000 genome	SIFT	Polyphen2 _HDIV	Mutation Taster
*GRIA2*	c.2308G > A (p.Ala770Thr)	AD	NE	NE	NE	Deleterious	Damaging	Disease_causing

In the gnomAD v2.1.1 database (https:// gnomad. broadinstitute. org/gene/ENSG00000120251? dataset = gnomad_r2_1), GRIA2 was highly constrained for missense variations (z-score: 4.56) and was intolerant to the loss of function (LoF, intolerance score: 1.00). Furthermore, the altered amino acid alanine, located in the ligand-gated ion channel domain, was highly conserved in multiple species (Fig. 2C) and may be crucial for protein stability and function. The SNV schematic in the reported *GRIA2* variant is shown in Fig. 2D, and the clinical characteristics of patients with developmental regression who carry the de novo* GRIA2* variant are summarized in our study (Table 2).

**Table 2:** The phenotype of patients with developmental regression who carry de novo* GRIA2* variant

Patient ID	P3	P4	P12	P16	Current
Variant	p.ASP611Asn	p.Gly609Arg	p.Pro286Leufs*14	p.Val647Leu	p.Ala770Thr
Study	Salpietro et al. 2019	Salpietro et al. 2019	Salpietro et al. 2019	Salpietro et al. 2019	Current
Gender/ Age onset	Early infancy/M	Infancy/F	2y/M	Early infancy/M	4y/F
DD	+	+	+	+	-
ID	+	+	+	+	-
ASD	+	-	+	n/a	+
Speech impairment	+	+	+	+	+
Walk	n/a	Mild dyspraxic gait	n/a	Unable	Normal
Seizures	No	No	No	Focal, tonic–clonic	No
Brain image	Normal	White matter changes	Normal	Mild cerebral atrophy	Normal
Other features	Obsessive- compulsive traits	Ataxic gait, dystonia	Obsessive- compulsive traits	n/a	Obsessive- compulsive traits, self-harm behaviors, psychiatric symptoms

### 3D protein modeling shows the structural change of variation in GRIA2

**Fig. 3:**
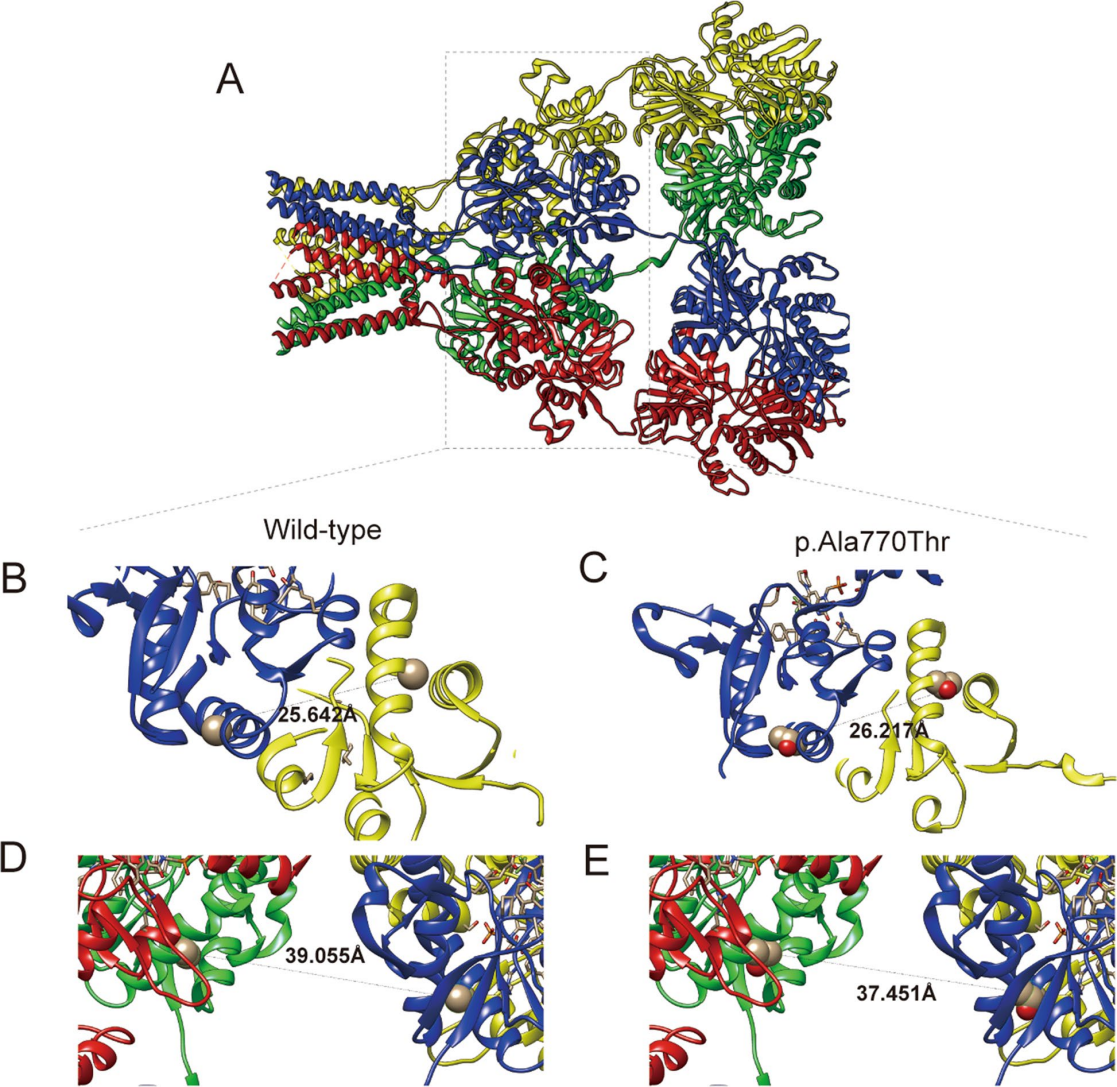
Protein modeling of AMPA receptor. **A.** Tetramer with four chains was visualized by USCF Chimera. Chains are indicated in different colors (A red, B blue, C yellow, and D green). **B, D** Wild-type alanine was highlighted by spheres. The distance of 770^th^ alanine was shown between chain B to C (**B**) and D (**D**) was shown. **C, E** Mutated alanine was highlighted by spheres. The distance of the 770^th^ threonine was shown between chain B to C (**C**) and D (**E**)

The structure of GluA2 was built to compare the mobility of mutated 770^th^ amino acid. The wild-type tetramer for the AMPA receptor was visualized by UCSF Chimera (Fig. 3A). The wild-type 770^th^ alanine and mutated 770^th^ threonine are highlighted. Minor variations in their backbones are shown. The distance between the wild-type and mutated amino acids in chains B and C was longer from 25.642 Å to 26.217 Å (Fig. 3B, C). In contrast, the distance between chains B and D was shorter, from 39.055 Å to 37.451 Å (Fig. 3D, E). Variant p.Ala770Thr was located in the ligand-gated ion channel domain, and these minor variations in protein structure may affect the transport of calcium ions. The stability of the GRIA2 protein structure was predicted by the mutation cutoff scanning matrix (mCSM), SDM, and DUET server. The scores (△△G) were -1.614, -2.62, and -1.824 kcal/mol, respectively, and all showed the destabilization of the p.Ala770Thr variant.

## Discussion and conclusions

To data, 23 SNVs in the *GRIA2* gene have been reported [8, 12], including 15 missense, two splicing, and five truncated variants. In all patients, seizures and abnormal brain structures were random. Twelve patients developed focal or tonic–clonic seizures, and all within six months of onset. Additionally, seven patients had abnormalities in their brain structure. All the patients had developmental delay and intellectual disabilities, but the ASDs, speech impairments, and motor delays were different. Recently, another clinical manifestation of childhood onset schizophrenia was found in a patient with a *GRIA2* truncated variation [12]. Since then, the phenotypes of *GRIA2* gene variants in the field of neurodevelopmental diseases have been expanded. However, based on the clinical characteristics of patients with different *GRIA2* variants, there does not seem to be a clear link between the localization of the variant and the clinical outcome.

Our report is the third study on the *GRIA2* variant in an individual onset of ASD and psychiatric symptoms. To the best of our knowledge, this is the first case characterized by atypical ASD with neuropsychiatric symptoms. She had an onset after four years of age and presented with stereotyped behaviors, language regression, and social interactions that were significantly reduced or absent, and visual hallucinations. These clinical characteristics match those observed in earlier studies and show the overlap of phenotypes related to *GRIA2* gene variants. ASD and schizophrenia are more likely to be seen in the same patients [13]. Various candidate genes for schizophrenia have also been reported to be related to ASD [14], which may be due to the core neurobiological processes that are likely common for the subsets of these two heterogeneous clinical groups. No seizures or brain structure abnormalities were found in our patient but she manifested as obvious regression in speech and social behaviors. It is interesting to note that behavior regression is not common in previous reports (Table 2). Only four patients had behavior regression and the age of onset was less than two years old. Regression with late-onset (more than three years old) as seen in our patient is rare [15]. Currently, there is no specific gene therapy or disease-modifying therapy for *GRIA2* variant disease. For patients with this gene variation, the main treatment is symptomatic therapy, such as antiepileptic drugs to control seizures[8], or treatment of psychiatric symptoms [12]. Epilepsy in patients with GRIA2 variants can be refractory. Psychiatric symptoms of a patient were reported to be partially relieved by clozapine treatment, followed by enhancement with lithium and aripiprazole [12]. Our patient was treated with risperidone, and her psychiatric symptoms (screams, irritability, visual hallucinations, etc.) was improved, but stereotyped behaviors and ASD-like manifestations still existed. Altered levels of GRIA2, which have been reported to be associated with the development of bipolar disorder [16], also provided clues to the psychiatric symptoms present in our patients. Development of drugs targeting GRIA2, like lithium[16], may be the direction for the treatment of related neurological diseases, and more cases need to be accumulated for clinical treatment exploration in the future.

The latest advances in ASD genetics, genomics, and transcriptomics have shown abnormal presynaptic and postsynaptic molecular assembly in synapses. In particular, due to changes in ASD risk genes, a series of presynaptic and postsynaptic proteins may be affected [17]. In recent years, in-depth research on the molecular basis and characteristics of ASD has revealed the potential role of AMPA receptor trafficking in ASD [18]. The AMPA receptor is composed of GluA1-4 subunits, in which GluA1/GluA2 heterotetramers are the most frequent combination in the forebrain [19]. All this evidence suggests that GRIA2 plays an important role in the development of the central nervous system. GRIA2 deficiency is a rare reason for neurodevelopmental disorders since only 28 patients with *GRIA2* variants have been reported [8]. The functional results showed that most of the variants will reduce mobility at the agonist binding site. Fifteen of the 23 variants are located in the ligand-gated ion channel domain (Fig. 2D), and the variant in our patient is also located in this region. Protein instability was shown in 3D protein structure and software prediction, and all these results indicated that protein function may be changed by this missense variant.

In summary, our study expands the spectrum of phenotypic variations of *GRIA2* and provides further evidence for the association between *GRIA2* variants and a late onset (four years old) of ASD symptoms with psychiatric symptoms. Furthermore, our case confirms the application of diagnostic WES in childhood ASD with psychiatric symptoms.

## Supplementary Information

**Additional file 1.** CARE-checklist.

## Data Availability

The datasets used and analyzed during the current study are available from the corresponding author on reasonable request.

## Abbreviations

ASDs: Autism spectrum disorders
CDC: Centers for Disease Control and Prevention
ECS: Editing complementary sequence
WES: Whole-exome sequencing
VEEG: Video electroencephalogram
MRI: Brain magnetic resonance imaging
DDST: Denver Developmental Screening Test
ABC: Autism behavior checklist
gnomAD: Genome aggregation database
ExAC: Exome aggregation consortium
*VUS*: Variant of uncertain significance
SNVs: Single nucleotide variations
