# Supplementary material for: Novel GRIA2 variant in a patient with atypical autism spectrum disorder and psychiatric symptoms: a case report
Source: BMC Pediatr. 2022 Nov 3;22:629. doi: 10.1186/s12887-022-03702-7 (PMC9632133; doi:10.1186/s12887-022-03702-7)
Supplement: Supplementary file 1 — Additional file 1. CARE-checklist. [file 12887_2022_3702_MOESM1_ESM.pdf]

CARE
case report guidelines

CARE Checklist of information to include when writing a case report

(cc) BY-NC-ND

Topic	Item	Checklist item description	Reported on Line
Title	1	The diagnosis or intervention of primary focus followed by the words "case report"	title, page 1,line 1
Key Words	2	2 to 5 key words that identify diagnoses or interventions in this case report, including "case report"	abstract, page 2, line 46
Abstract (no references)	3a	Introduction: What is unique about this case and what does it add to the scientific literature?	background, page1
	3b	Main symptoms and/or important clinical findings	case presentation, page 1
	3c	The main diagnoses, therapeutic interventions, and outcomes	case presentation, page 1
	3d	Conclusion—What is the main "take-away" lesson(s) from this case?	conclusion, page 1
Introduction	4	One or two paragraphs summarizing why this case is unique (**may include references**)	background, page 2
Patient Information	5a	De-identified patient specific information.	case report , paragraph(p) 1
	5b	Primary concerns and symptoms of the patient.	case report, p2
	5c	Medical, family, and psycho-social history including relevant genetic information	case report, p1
	5d	Relevant past interventions with outcomes	case report, p3
Clinical Findings	6	Describe significant physical examination (PE) and important clinical findings.	case report, p2; genetic finding, line121-142
Timeline	7	Historical and current information from this episode of care organized as a timeline	case report p1-3
Diagnostic Assessment	8a	Diagnostic testing (such as PE, laboratory testing, imaging, surveys).	case report p2, genetic finding line121-142
	8b	Diagnostic challenges (such as access to testing, financial, or cultural)	genetic finding, line 121-142
	8c	Diagnosis (including other diagnoses considered)	case report p2, genetic finding line121-142
	8d	Prognosis (such as staging in oncology) where applicable	case report, p3
Therapeutic Intervention	9a	Types of therapeutic intervention (such as pharmacologic, surgical, preventive, self-care)	case report, p3
	9b	Administration of therapeutic intervention (such as dosage, strength, duration)	case report, p3
	9c	Changes in therapeutic intervention (with rationale)	case report, p3
Follow-up and Outcomes	10a	Clinician and patient-assessed outcomes (if available)	N/A
	10b	Important follow-up diagnostic and other test results	N/A
	10c	Intervention adherence and tolerability (How was this assessed?)	N/A
	10d	Adverse and unanticipated events	N/A
Discussion	11a	A scientific discussion of the strengths AND limitations associated with this case report	discussion, p1
	11b	Discussion of the relevant medical literature **with references**.	discussion, page 4
	11c	The scientific rationale for any conclusions (including assessment of possible causes)	discussion, p2-3
	11d	The primary "take-away" lessons of this case report (without references) in a one paragraph conclusion	discussion, p4, line201-204
Patient Perspective	12	The patient should share their perspective in one to two paragraphs on the treatment(s) they received	N/A
Informed Consent	13	Did the patient give informed consent? Please provide if requested	**Yes** ☑ **No** ☐
